# Molecular, Metabolic, and Subcellular Mapping of the Tumor Immune Microenvironment via 3D Targeted and Non-Targeted Multiplex Multi-Omics Analyses

**DOI:** 10.3390/cancers16050846

**Published:** 2024-02-20

**Authors:** Sammy Ferri-Borgogno, Jared K. Burks, Erin H. Seeley, Trevor D. McKee, Danielle L. Stolley, Akshay V. Basi, Javier A. Gomez, Basant T. Gamal, Shamini Ayyadhury, Barrett C. Lawson, Melinda S. Yates, Michael J. Birrer, Karen H. Lu, Samuel C. Mok

**Affiliations:** 1 Department of Gynecologic Oncology and Reproductive Medicine, The University of Texas MD Anderson Cancer Center, Houston, TX 77030, USAkhlu@mdanderson.org (K.H.L.); 2 Department of Leukemia, The University of Texas MD Anderson Cancer Center, Houston, TX 77030, USA; dstolley@mdanderson.org (D.L.S.); avbasi@mdanderson.org (A.V.B.); jagomez2@mdanderson.org (J.A.G.); 3 Department of Chemistry, The University of Texas at Austin, Austin, TX 78712, USA; erin.seeley@utexas.edu; 4 Pathomics, Inc., Toronto, ON M4C 3K2, Canada; trevor@pathomics.io (T.D.M.); shamini@pathomics.io (S.A.); 5 Department of Anatomical Pathology, The University of Texas MD Anderson Cancer Center, Houston, TX 77030, USA; 6 Department of Pathology and Laboratory Medicine, The University of North Carolina, Chapel Hill, NC 27599, USA; 7 Winthrop P. Rockefelle Cancer Institute, The University of Arkanasas for Medical Sciences, Little Rock, AR 72205, USA

**Keywords:** ovarian cancer, atypical endometrial hyperplasia, Stereo-seq, mass spectrometry imaging, microbiome, tumor microenvironment, 3D spatial multi-omics

## Abstract

**Simple Summary:**

A tumor tissue is composed of not only cancer cells but also other cell types and microorganisms that communicate among themselves in a three-dimensional (3D) space to support cancer cell growth. Using two different gynecologic tumor tissue samples, we integrated multiple new techniques using a suite of newly developed analytical methods to simultaneously identify expression of genes, metabolites, and proteins in single tissue ‘voxels’. These tissue voxels contain cells, genes from those cells and microorganisms, and the stromal context of proteins (collagen), glycans, metabolites, and peptides, all identified in the 3D space within a tumor tissue. We have successfully demonstrated different arrays of analytes expressed by cancer cells and their neighboring cells in different regions of the tumor tissue. Understanding how cancer cells communicate with other cell types in the 3D space of the tumor tissue will allow for the identification of new therapeutic targets for the treatment of these diseases.

**Abstract:**

Most platforms used for the molecular reconstruction of the tumor–immune microenvironment (TIME) of a solid tumor fail to explore the spatial context of the three-dimensional (3D) space of the tumor at a single-cell resolution, and thus lack information about cell–cell or cell–extracellular matrix (ECM) interactions. To address this issue, a pipeline which integrated multiplex spatially resolved multi-omics platforms was developed to identify crosstalk signaling networks among various cell types and the ECM in the 3D TIME of two FFPE (formalin-fixed paraffin embedded) gynecologic tumor samples. These platforms include non-targeted mass spectrometry imaging (glycans, metabolites, and peptides) and Stereo-seq (spatial transcriptomics) and targeted seqIF (IHC proteomics). The spatially resolved imaging data in a two- and three-dimensional space demonstrated various cellular neighborhoods in both samples. The collection of spatially resolved analytes in a voxel (3D pixel) across serial sections of the tissue was also demonstrated. Data collected from this analytical pipeline were used to construct spatial 3D maps with single-cell resolution, which revealed cell identity, activation, and energized status. These maps will provide not only insights into the molecular basis of spatial cell heterogeneity in the TIME, but also novel predictive biomarkers and therapeutic targets, which can improve patient survival rates.

## 1. Introduction

The complex ecosystem of a solid tumor is composed of tumor cells, immune cells, stromal cells, fibroblasts, extracellular matrix (ECM), blood vessels, and intratumoral microbiota, which constitute the tumor–immune microenvironment (TIME) [1,2]. Dynamic and bidirectional interactions occur among various cell types through direct cell–cell interaction or communication signals such as secreted proteins, glycans, metabolites, and microvesicles such as exosomes modulate the malignant phenotype of the tumor cells so that they can survive, proliferate, and modulate therapeutic efficacy in the oxygen- and nutrient-limiting TIME [2,3,4].

Cellular and molecular reconstruction of the TIME of a solid tumor is of great significance to the field of cancer research and will have an immediate impact in the clinical management of cancer patients [5]. For example, public datasets such as data generated from the Cancer Genome Atlas (TCGA) projects demonstrate how these data provide new insights into cancer research. TCGA projects have provided results, which have been referenced extensively, and their data have been used to generate many new projects and hypotheses [6]. However, TCGA mainly uses bulk tissue samples, and therefore the data cannot be used to address key questions including how intertumoral and intratumoral heterogeneity develop, how tumor cells and various stromal cell types interact in the TIME, and how certain histological patterns within tumors such as the location of activate CD8+ T cells play an important role in modulating the malignant phenotypes of tumor cells [7,8,9]. Recent single-cell RNA sequencing (scRNAseq) projects and datasets from various tumor types allow the study of tumor heterogeneity [10,11,12,13]. However, the initial tissue dissociation step disrupts any three-dimensional spatial context, thus again lacking any information about cell–cell or cell-ECM interactions. 

Multiple spatially resolved omics platforms including spatial transcriptomics (ST), imaging mass cytometry (IMC), mass spectrometry imaging (MSI), and multiplex sequential immunofluorescence (seqIF) have recently been developed, which allow for both targeted or non-targeted profiling of mRNAs, metabolites, and proteins in both frozen and formalin-fixed paraffin embedded (FFPE) tissue sections with spatial context [14,15,16,17,18,19,20]. Data generated by these platforms have provided an immediate identification of new signaling networks, delineate crosstalk between stromal and epithelial components, characterize the nature and function of immune infiltrates, and determine how these change with anatomical location, treatment response, and patient survival [21,22,23,24]. However, most of these platforms, particularly those using non-targeted approaches, do not provide true single-cell level resolution. For example, each spatial transcriptomics platform spot contains multiple cell types, and deconvolution using parallel scRNA-seq analyses of the same specimen are required to re-assign and adjust the spatial transcriptomics data [17,22]. Non-targeted MSI data needs to be integrated with IMC for single-cell metabolomics profiling [25]. In addition, most of the experiments have been performed on a single histological tissue section of a particular tumor sample in which cellular interaction can only be observed in 2D space. Interaction with neighboring cells in the adjacent histological planes, which may be located closer than those observed in the 2D space, have not been considered. Three-dimensional (3D) multi-omics mapping of the TIME has just only begun in recent studies [26]. Finally, it remains a challenge to integrate data generated by multiple spatially resolved omics platforms, which will allow for discovering novel cell–cell or cell-ECM crosstalk signaling networks and facilitating hypothesis generation with mechanistic insights.

Here, we describe a novel pipeline for the construction of a 3D spatial atlas with subcellular resolution using FFPE sections obtained from a high-grade serous ovarian cancer (HGSOC) and atypical endometrial hyperplasia (AEH) tissue, and multiple spatially resolved omics platforms including the newly developed spatial enhanced resolution omics-sequencing (Stereo-seq), mass spectrometry imaging (MSI), and multiplex sequential immunofluorescence (seqIF). We also demonstrate the feasibility of using the pipeline to integrate data from these platforms to identify cell–cell crosstalk networks among various cell types in the 3D space of the TIME. The pipeline can be applied to large patient cohorts to identify novel spatially resolved predictive and prognostic biomarkers, which can be used to develop new therapeutic agents and strategies in cancer treatment.

## 2. Materials and Methods

### 2.1. Patient Samples

Two paraffin-embedded tumor tissue samples were used in this study. One was obtained from a patient with stage IIIC HGSOC. The other one was obtained from a patient with atypical endometrial hyperplasia (AEH). They were collected from previously untreated patients undergoing primary cytoreductive surgery and hysterectomy for HGSOC and AEH, respectively. All samples and clinical data were collected with the approval of MD Anderson’s Institutional Review Board. Clinicopathologic characteristics of utilized samples are shown in Table S1.

### 2.2. Sample Preparation for 3D Multi-Omics Analyses

**Figure 1:**
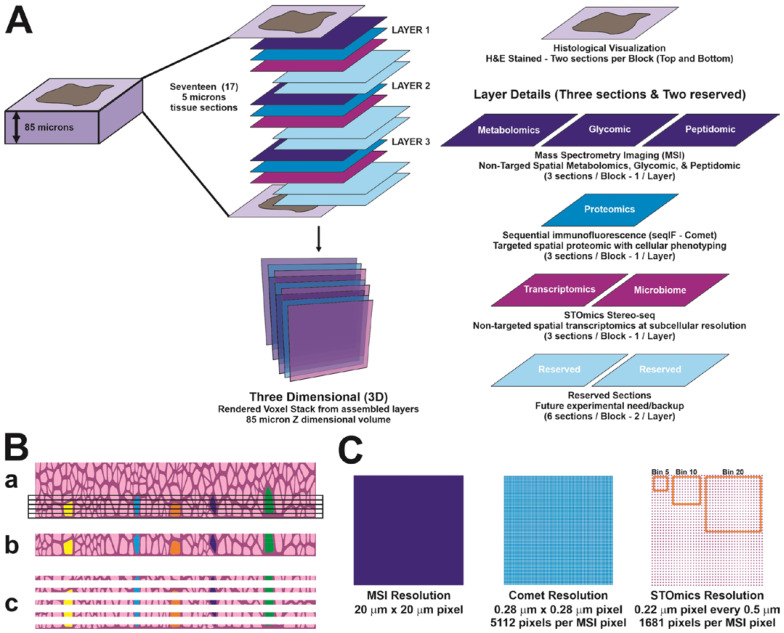
Schematic diagram summarizing the 3D targeted and non-targeted multiplexed multi-omics workflow used to dissect the tumor immune microenvironment. (**A**) For each sample, 5 mm serial FFPE sections from an 85 mm thick tissue block were cut and deposited onto slides specific for each platform. The first and the last sections were stained with H&E for histological evaluation. Every third section was used for non-targeted metabolomics, glycan, and tryptic peptide analysis (by mass spectrometry imaging, MSI), targeted proteomics (by multiplexed sequential immunofluorescence, COMET) or non-targeted spatial transcriptomics (Stereo-seq by STOmics) analyses. (**B**) Rationale for using serial tissue section for analysis with different modalities. The same cell (average diameter 20 microns) transverses multiple sections (5 microns each, **a**–**c**), equate to 4–5 sections. (**C**) Resolution of the three different modalities used in the study, by comparing pixel dimensions.

For each tissue block, seventeen 5 µm serial FFPE sections from an 85 µm-thick tissue block were cut and deposited onto slides specific for each platform. The first and the last sections were stained with hematoxylin and eosin (H&E) for histological evaluation as described in Figure 1 (in Section 3). Sections were cut to a step size of 5 µm, and every third section was used for non-targeted metabolomics, glycan, and tryptic peptide analysis (by mass spectrometry imaging, MSI), targeted proteomics (by multiplexed seqIF, COMET) or non-targeted Stereo-seq (by STOmics) analyses. To generate a 3D atlas, 3 sections per specimen as shown in Figure 1 were evaluated for each of the three platforms. Sections for Stereo-seq analysis were deposited onto a DNA Nanoball (DNB)-patterned array chip (STOmics, San Jose, CA, USA). Sections for MSI and COMET analyses were deposited onto superfrosted microscopic glass slides (Fisherbrand, Toronto, ON, Canada).

### 2.3. Sequential Immunofluorescence Staining (seqIF)

SeqIF was performed using the COMET instrument (Lunaphore Technologies SA, Tolochenaz, Switzerland) as previously described [27]. In brief, FFPE tissue slides were deparaffinized in xylene followed by rehydration in a graded alcohol series and blocked with 3% hydrogen peroxide for 10 min. Antigen retrieval was performed with EZ-AR2 Elegance buffer (BioGenex, Fremont, CA, USA) at 107 °C in an EZ-Retriever system V.3 (BioGenex, Fremont, CA, USA) for 15 min. The processed slides were then transferred to a Multistaining Buffer (BU06, Lunaphore, Tolochenaz, Switzerland) bath until use [27]. The microfluidic chip (9 × 9 mm imageable area) was clamped against the FFPE tissue section on a standard microscope slide forming a closed reaction chamber. The reagents were delivered through microfluidic channels under highly controlled conditions. Automated multiplex sequential immunofluorescence staining and imaging was performed on the COMET platform (Lunaphore Technologies, Switzerland). Slides underwent 10 cycles of iterative staining and imaging, followed by an elution of the primary and secondary antibodies at each cycle. Primary antibodies were diluted to desired concentrations based on preliminary titration tests to optimize signal-to-noise ratio (see Tables S2 and S3) in multistaining buffer (BU06, Lunaphore Technologies) with 3% BSA (Millipore Sigma, Burlington, MA, USA) and 1% horse serum (Millipore Sigma). Secondary anti-rabbit/anti-mouse Alexa Fluor 555 (Invitrogen, Waltham, MA, USA) and Alexa Fluor 647 (Invitrogen) were used at 1:200 and 1:400 dilutions in multistaining buffer, respectively regardless of species reactivity (see Table S4). 4′,6-diamidino-2-pheynlindole DAPI (Thermofisher, Waltham, MA, USA) was used either alone, or in conjunction with secondary antibodies, at a 1:2000 dilution in multistaining buffer.

The 20-plex protocol template was generated using the software program COMET Control, and reagents were loaded onto the device to perform the multiplex sequential immunofluorescence protocol. Images were taken by the integrated epifluorescent microscope at 20× magnification using DAPI (exposure time, 80 ms), TRITC (exposure time, 400 ms), and Cy5 (exposure time, 200 ms) channels for every cycle with an imaging area of 9 × 9 mm. Initial images were captured for autofluorescence subtraction during image processing post-acquisition and to provide DAPI nuclear counterstaining. Primary antibody incubation was carried out for 4 or 8 min for each cycle based on prior optimization (see Tables S2 and S3), and all secondary antibodies were incubated for 2 min (Table S4). Antibodies were then eluted following each cycle for 4 min. The seqIF protocol in COMET resulted in a multi-layer OME-TIFF file where the imaging outputs from each cycle were stitched and aligned. COMET OME-TIFF contains DAPI image, intrinsic tissue autofluorescence in TRITC and Cy5 channels, and a single fluorescent layer per marker. Images were exported from COMET after background subtraction. 

#### 2.3.1. Sequential Immunofluorescence Data Analysis

Image analysis was performed using Visiopharm image analysis software version 2023.09 x64 (Visiopharm Inc., Hoersholm, Denmark). Fluorescent images of layers 1, 2, and 3 for each sample were first aligned to the corresponding MSI images utilizing the Tissuealign module to obtain a 3-dimensional image. The seqIF layer was used for tissue segmentation to separate different tissue areas. For the HGSOC sample, tumor and stroma areas were identified based on expression of keratin 8/18 and Col1A1, respectively. Subsequently, the tumor and stroma areas were each eroded by 50 μm to obtain an interface width of approximately 100 μm. For the AEH sample, tissue segmentation was performed utilizing pan-keratin, CD10 and αSMA markers to define glandular epithelium, glandular stroma, and muscle regions, respectively. The space inside each glandular epithelium was labelled glandular lumen. After tissue segmentation, cell boundaries were determined by a pretrained machine learning algorithm that used DAPI channel to automatically identify nuclei and cells. Identified cells were then phenotyped using Visiopharm’s unbiased autoclustering module using only the top 20% of pixel values per cell.

#### 2.3.2. 3D Reconstruction of seqIF Images

Three regions of interest were selected and subsequently extracted from the COMET background subtracted multi-layer OME-TIFF files for 3D reconstructions. In brief, extracted regions were imported into QuPath software version 0.5.0 and aligned using Warpy software version 0.3.0 [28,29]. The second of the three tissue sections was used as the base image with the first and third sections overlaid and manually aligned using the Image Combiner Warpy interactive alignment tool with nearest neighbor interpolation [29]. Subsequently, alignment was performed using affine transformation registration and image intensity alignment. Fine manual adjustments were made with interactive alignment to ensure tissue structure between samples was aligned before creating the combined overlay image. The combined and aligned image was then exported from QuPath as original pixels to TIFF. 

The combined and aligned TIFF image was converted and imported into Imaris version 10.1.10 (Bitplane, Belfast, UK) using ImarisFileConverter. Fluorescent channels were re-assigned to each marker, and each section was exported as a separate file. The three resulting files for each region of interest were then combined in Imaris as slices, the voxel size adjusted to Z = 0.23 μm (equal to XY voxel dimensions), and the combined 3D image was resampled maintaining X/Y aspect ratio in 3D to generate 85 slices for visualization. The resampled 3D image was cropped to 575 × 575 × 19.6 μm (2500 × 2500 × 85 pixels) to remove areas that were missing signal in a slice due to prior alignment in QuPath.

### 2.4. Stereo-Seq Analysis

Spatially resolved transcriptomes were generated from the HGSOC and AEH tissue samples by Stereo-seq following established protocols. In brief, paraffin sections, cut to a thickness of 5 μm, were mounted on Stereo-seq N transcriptomics chips (Cat#210CN114, STOmics). The Stereo-seq procedure adhered to the vendor’s manual and prior publications [30,31]. Briefly, the tissue section on the Stereo-seq chip (1 cm × 1 cm) underwent a drying process for 3 h at 42 °C, followed by overnight drying (up to 48 h) at 37 °C. Paraffin was melted at 60 °C for 1 h, deparaffinized in xylene substitute and ethanol, and then subjected to de-crosslinking using the STOmics reagent kit (Cat# 211KN114, STOmics). Fixation in pre-cooled methanol (Cat# 34860, Sigma) for 20 min at −20 °C ensued. Post-fixation, the Stereo-seq chip was air-dried, and the tissue section was incubated in permeabilization buffer (Cat# 211SN114, STOmics) for 30 min at 37 °C. After permeabilization, FFPE Dimer mix (Cat# 211SN114, STOmics) was added and incubated at 25 °C for 1 h. Captured RNAs were reverse-transcribed and ligated onto the transcriptomics chip surface at 42 °C overnight. Subsequently, cDNAs were released from the chip using the transcriptomics reagent kit (Cat# 211KN114, STOmics). After size selection, amplification, and purification, cDNA concentration was quantified using the Qubit dsDNA HS assay kit (Cat# Q32854, Invitrogen). Library construction utilized 20 ng of cDNA from each sample with the library preparation kit (Cat# 111KL114, STOmics) and subsequent DNB (DNA Nano Ball) generation. The DNBs were sequenced on the DNBSEQ T7 sequencing platform with 91 cycles of Read 1 and 100 cycles + 10 bp barcode of Read 2 (Cat#940-000838-00, Complete Genomics, San Jose, CA, USA).

#### 2.4.1. Stereo-Seq Raw Data Processing

Fastq files were generated using a DNBSEQ-T7 sequencer. CID were MID are contained in the read 1 (CID: 1–25 bp, MID: 26–31 bp) while the read 2 consist of the cDNA sequences. CID sequences on the first reads were first mapped to the designed coordinates of the in situ captured chip achieved from the first round of sequencing, allowing 1 base mismatch to correct for sequencing and PCR errors. Reads with MID containing either N bases or more than 2 bases with quality score lower than 10 were filtered out. CID and MID associated with each read were appended to each read header. Retained reads were then aligned to the reference genome (hg38) using STAR [32] and mapped reads with MAPQ > 10 were counted and annotated to their corresponding genes). UMI (Unique Molecular Identifier) with the same CID and the same gene locus collapsed, allowing 1 mismatch to correct for sequencing and PCR errors. Non-host reads were then collected and MID counts for proportion of microbes were calculated. Finally, this information was used to generate a CID-containing expression profile matrix.

#### 2.4.2. Stereo-Seq Data Processing

Analysis of Stereo-seq data was conducted in a conda environment using stereopy/1.1.0 and scanpy/1.9.6. Raw data from above were loaded onto Python using the stereopy package one sample at a time forming a list. Quality control measurements were performed using default settings found in the cal_qc() function from stereopy where cells with minimum genes = 3, minimum genes by counts = 3 and mitochondrial percentage >20% being excluded from the analysis. Next, we merged the 3 sections from each patient, using the data_helper.merge() function from stereopy, forming 2 patient batches. Merging was necessary to project the results back onto the same gene clusters, rather than leave the three sections to cluster independently. This was followed by normalization and log1p transformation over each batch using the standard normalization/transformation settings from stereopy. Batch PCA (Principal Component Analysis) and data integration (using default harmony settings from stereopy) was carried out using all genes and principal components respectively. The standard settings for the ‘find neighbors’ function and Leiden clustering were applied using the relevant functions from stereopy, over each batch corrected patient set. Following this, uniform manifold approximation and projection (UMAP) was applied to dimensionally compress the gene information into 2D plots, using default settings from stereopy. The UMAP coordinates were plotted colored by Leiden cluster, to indicate the relationship between the different clusters. Each batch was then converted to an AnnData object and the rank_genes_groups function from scanpy was applied, over each sample in a batch, using the ‘Wilcoxon’ statistical analysis method. Finally, the spatial coordinates (in x,y) for individual sections, alongside the combined Leiden coordinates, and gene expression values for samples by batch were combined into a single dataframe. The top genes were identified for each Leiden cluster by selecting for differentially expressed genes (from rank_genes_groups() function) that had significance of *p* < 0.01 and log2foldchange > 1 and that were shared across the sections in a batch. The AnnData objects per each 3 sections were converted into data frames and exported as csv files, which were then converted from a list of pixel values to a 2D map, in which individual channels held a unique index value that corresponded to a particular Leiden cluster x and y value. This ‘virtual image’ was then warped using affine transformation to align it with the COMET and MSI datasets. From there, the virtual image was used in similar ways to a ‘look up table’ or a hash in a database, a unique value that corresponds to a particular spot within the tissue. In this fashion, a unique tissue region was identified by the particular patterns that showed up in the Leiden gene space, and was further identified by its appearance in ‘protein space’, namely the H&E and multiplex immunofluorescence image, which contain pathologist-identified regions of distinct tissue morphology, and protein expression denoting common tissue features such as stroma, epithelium (including tumor), and other tissue compartments. 

### 2.5. MSI Analysis

#### 2.5.1. MSI Sample Preparation

Prior to metabolite analysis, sections were deparaffinized with xylene, 2 × 3 min, with no further rehydration. Fiducial points were etched onto the slides with a diamond scribe, and images were acquired of the sections using an Epson Perfection V600 Photo flatbed document scanner (Epson US, Los Alamitos, CA, USA) at 4800 dpi. All matrix and enzyme application were carried out with an HTX M5 Robotic Reagent Sprayer (HTX Technologies, LLC, Chapel Hill, NC, USA). Full details of spraying conditions for each spray application are summarized in Table 1. Briefly, sections were coated with 10 mg/mL 1,5-diaminonaphthalene (DAN) matrix in 50% ACN for metabolite analysis. After metabolite imaging, the matrix was removed with 100% ethanol, and the sections were rehydrated with grade ethanol. Antigen retrieval was performed in a Biocare Medical Decloaking Chamber™ NxGen (Biocare Medical, Pacheco, CA, USA) in 100 mM Tris at pH 9 for 20 min at 95 °C. For in situ release of N-linked glycans, the sections were coated with PNGaseF (Bulldog Bio, Portsmouth, NH, USA) using the HTX Sprayer, and the slides were incubated in a humidity chamber for 2 h at 37 °C [33]. After PNGaseF digestion, the sections were coated with 10 mg/mL α-cyano-4-hydroxycinnamic acid (CHCA) matrix in 70% ACN, 0.1% TFA, 10 mM ammonium phosphate using the HTX Sprayer. After glycan imaging, matrix was again removed with ethanol and the rehydration and antigen retrieval repeated. The sections were then coated with trypsin using the HTX Sprayer and the slides were incubated in a humidity chamber for 4 h at 37 °C. After tryptic digestion, the slides were again coated with CHCA matrix using the HTX Sprayer. Finally, after tryptic peptide image acquisition, matrix was removed using ethanol and the sections were hematoxylin and eosin stained using standard protocols. Digital images were acquired of the sections at 20× magnification using a Hamamatsu NanoZoomerSQ Digital Slide Scanner (Hamamatsu Photonics, Bridgewater, NJ, USA).

#### 2.5.2. Mass Spectrometry Imaging

All mass spectrometry images were acquired on a Bruker timsTOF fleX QTOF mass spectrometer (Bruker Daltonics, Billerica, MA, USA) at 20 µm resolution: metabolites in negative ion mode, glycans and metabolites in positive ion mode. Methods were optimized for each analysis using timsControl 3.1 and acquisition parameters are summarized in Table 2. FlexImaging 7.0 was used to align each slide to the optical image acquired on the Epson scanner using the etched fiducial points. The aligned image was then used to guide data collection from the tissue sections. Prior to each image acquisition, the instrument was mass-calibrated using red phosphorus.

After collection, data files were loaded into SCiLS Lab 2024a Pro (Bruker, Daltonics, Billerica, MA, USA) for visualization. Peaks from each imaging dataset were manually picked, excluding known matrix peaks and non-monoisotopic peaks. Metabolites and glycans were putatively identified using the SCiLS MetaboScape 2023b plugin. Metabolites were searched against the human metabolite database as [M-H]^−^, [M]^−^, and [M+Cl]^−^ primary ions, and glycans were searched as [M+H]^+^, [M+Na]^+^, and [M+K]^+^ primary ions. Images were exported from SCiLS as .imzML files for further analysis and integration with other imaging modalities. 

### 2.6. Integration of Multi-Omics Platforms

A key element of our approach was to base the alignment between the modalities against the STOmics reference frame, as the data output from the standard STOmics workflow included files that could be converted into AnnData objects, for subsequent analysis using the scverse pipelines. Given the choice of coordinate system with the STOmics data, we utilized one of the elements of the structure of the data, the capacity to ‘bin’ individual Stereo-seq spots together into ‘bins’ of increasing dimension. There are actually interesting parallels between this and the ‘pyramid’ structure of pathology images, such as H&E or COMET, in that the high resolution pixel data is overlaid with downsampled tiles at lower resolutions, allowing a lower resolution overview of different scales of tissue morphology and structure. The Leiden cluster at bin 50 could be considered roughly equivalent to a 5× magnification on a tissue microscope, able to visualize broad tissue structures, but not necessarily more highly resolved finer features. The bin50 downsampling also permitted us to integrate enough genes across those bins to achieve reasonable computational times for Leiden gene clustering, as well as avoid the appearance of too ‘noisy’ of a Leiden cluster map, given that the smaller bins may also contain less coverage than larger bins. 

Once the Leiden cluster alignment was complete for COMET datasets, subregions were then accessed within the tissue, using the Tissue Segmentation and Cell Segmentation, and cellular phenotypes, identify (in ‘protein-space’) unique biological entities present in the region. Downstream analysis could include more in-depth assessment of spatial neighborhood analysis and other aspects of the tissue architecture, but from here we evaluated at a baseline the correlation between cell phenotypes, tissue structure, and molecular readouts with the Leiden cluster ‘voxel’, comprised of a set of x and y coordinates within which the bin50 clustering was performed. Evaluating each bin50 region as a ‘Leiden cluster voxel’, and then looking at the distribution of gene expression values, cell phenotypes, and MSI data, as an aggregate of bin50 ‘voxels’ present within each Leiden cluster, permitted us to interrogate the molecular information present at the same x and y coordinate across modalities. Likewise, Leiden gene cluster to mass spectrometry alignment permitted us to interrogate peaks that showed discrete tissue localization, and identify areas of alignment between these two disparate data sources.

In addition to alignment using the Leiden gene cluster maps, the MSI data were also directly aligned with the COMET datasets within the Visiopharm software version 2023.09 x64 TissueAlign feature, which permitted us to identify tissue regions and phenotypes that matched features seen in the MSI data, in order to validate that the alignment through Leiden matched with a direct alignment between the protein and mass spectrometry analysis. 

## 3. Results

### 3.1. Sequential Immunofluorescence Analysis

**Figure 2:**
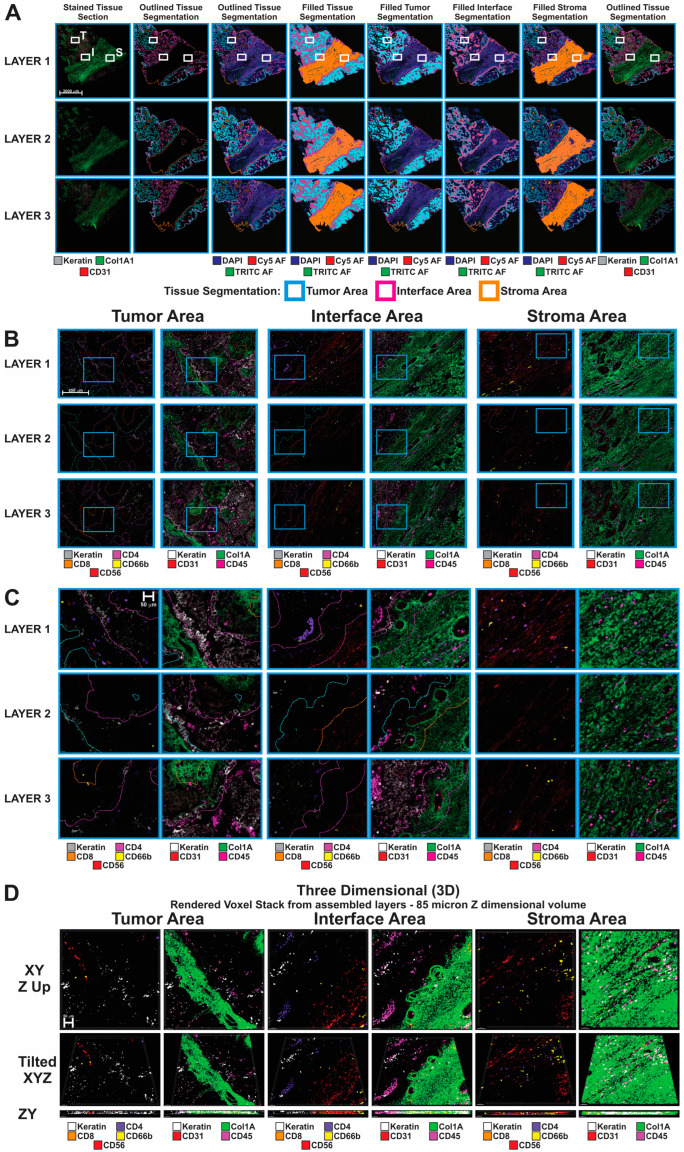
Multiplex sequential immunofluorescence analysis on HGSOC sample. (**A**) Whole tissue images overlaid with tissue segmentation mask created based on Keratin8/18 and COL1A1 markers in Visiopharm software for each layer. White boxes represent chosen ROIs for tumor (T), stroma (S) and interface (I) areas shown in panel (**B**). Scale bar is 3000 µm. (**B**) Chosen ROIs from panel (**A**), highlighting corresponding marker expression in tumor, stroma and interface areas. Blue squares represent chosen ROIs shown in panel (**C**). Scale bar is 450 µm. (**C**) Chosen ROIs from panel (**B**), showing differential expression of main immune markers in tumor, stroma and interface areas. Scale bar is 50 µm. (**D**) 3D reconstruction through rendered voxel stack from assembled layers 1–3. Scale bar is 50 µm.

Multiplex sequential immunofluorescence analysis on the 3 FFPE sections prepared from an HGSOC, and an AEH sample as shown in Figure 1 was performed using the COMET instrument (Lunaphore, Tolochenaz, Switzerland) as previously described [26]. A panel of 20 protein markers was designed for the HGSOC or AEH tissue samples. These markers were detected simultaneously by specific antibodies on a single tissue section (Tables S2 and S3). Figure 1B demonstrates that many cells traverse multiple sections in a tumor block given standard 3–5 micron sectioning. It is the foundation of this concept that allows the multi-modality approach to function. Figure 1C is a visual demonstration of the various resolutions of the technologies employed, where COMET features the highest resolved value, as no binning or unionization of the pixel values are needed. The STOmics is classically binned, but even with the binning typically performed is higher resolved than its closest competing assay, VisiumHD. Image data were successfully generated and applied for tissue and cell segmentation, which were used for the analysis of Stereo-seq aand MSI data generated from adjacent sections. For the HGSOC samples, we were able to define tumor, stroma, and interface areas using Keratin8/18 and Col1A as markers (Figure 2A). Regions of interest (ROI) were selected from the tumor enriched, stroma enriched and interface area for further analysis using additional stromal cell markers including those specific for immune cells and endothelial cells (Figure 2B,C). For the AEH samples, glandular epithelium, stroma, and luminal areas were identified using Pan-keratin and CD10 as markers for tissue segmentation (Figure S1), while muscle cells in the myometrium area were segmented based on αSMA expression (Figure S1).

The Lunaphore COMET SeqIF data is understated here as we used it as a screening and naming tool to identify tissue landmarks and cell phenotypes. Additional and more traditional analysis including neighborhood analysis is possible and will be performed but would require a larger cohort of images. Clinical correlation is also an additional analytical step, and this correlation could be continued across the additional imaging omics methods presented, however this adds in complexity and the purpose of this manuscript is to demonstrate the initial integration of the three technologies. The imaging produced from the seqIF is the cornerstone of the other imaging modalities, due to its ability to capture both morphological tissue patterns at high resolution, as well as molecular information about specific cell types present within the tissue.

In agreement with the findings presented in Kuett et al. [26], we demonstrated that features, adjacent cells, vessels, etc., are closer in the Z dimension than in the X and Y dimensions. This speaks to the fact that clustering or neighborhoods are not two-dimensional objects they occupy a three-dimensional space. This means that many of the findings related to the relevance of immuno-supportive or immuno-suppressing may not be found in the same plane, but in the planes above or below. Even in the limited tissue examples used in this study, the complexities of the tumor, interface, and stromal compartments is visible in Figure 2B,C. With closer inspection, one can see that in the tumor sample of Figure 2C, CD66b positive cells were found in the upper left quadrant within a small space of each other in layer 1 and layer 3. When we consider the sections between these CD66b positive cells are within ~20 microns of each other, significantly closer as there are no other CD66b positive cells in layer 1. Reflecting on the second tumor area, this is also very visible with the CD45 positive immune population as it appears to cluster in the keratin positive tumor area. The Interface area, which involves CAFs, immune cells, and tumor cells is the most complicated and interesting area as we previously described [21,22]. The ability to align the datasets produced in this paper permits us to start asking advanced questions to further interrogate this important tissue region (Figure 2D).

Figure S1 of the AEH precancerous lesions is much more complicated due to the complexity of the lesion. Of interest is the observation that the lesions, particularly in the luminal area, are filled with metabolites. This would serve as a metabolomics reserve for this area’s tissue development. The same observations are possible when considering the CD45 positive immune cells or the CD10 positive cells in ROI3. Understanding how this area develops is critical to early cancer detection. The aforementioned observations and measurements are possible in a series of two-dimensional images or even in a series of data tables that have a shared cartesian coordinate system of X, Y, Z. However, much of biology is from observation; seeing is believing in many cases. Rendering these areas in 3-dimensional space gives the observer the ability to dive into and around the cells in their environment and make the observations, which can then be hypothesized and tested. This is the critical importance behind collecting sections and holding them in reserve as suggested in Figure 1. This allows for reflection on the initial data collection and to have follow-up studies performed on proximal samples for clarification and extension using these research methods.

### 3.2. Generation of Robust Spatially Resolved Transcriptomic Profiles Using the Stereo-Seq Chip

The Stereo-seq N transcriptomics chips (1 cm × 1 cm) (STOmics) were used to generate spatially resolved transcriptomic profiles from 3 FFPE sections prepared from HGSOC and AEH samples as shown in Figure 1. The DNB patterned chip uses unique molecular identifiers (MIDs) and poly N sequence-containing oligonucleotides ligated onto each spot on the chip through hybridization with an oligonucleotide sequence containing the coordinate identity (CID), which allows capturing not only human coding and non-coding RNA but also microbiome RNAs. In the 3 sections of HGSOC sample, stereo-seq captured mean gene type counts ranging on average from 59.44 per 100 μm^2^ (bin 20, 10 μm × 10 μm) to 97.85 per 100 μm^2^ (bin 20), equivalent to the size of ~1 median size cell (Table 3 and Table 4); while in the 3 sections of AEH sample, stereo-seq captured mean gene type counts ranging on average from 91.64 per 100 μm^2^ (bin 20) to 102 per 100 μm^2^ (bin 20). Mitochondria transcripts were found to be less than 2% in all the sections. Total microbiome transcript counts ranged from 24,980 to 104,870 (Table 3). They include bacteria, fungi, and viruses, with a majority belonging to the Actinomycetia and Gammaproteobacteria classes (Figures S3 and S4).

#### High Levels of Tumor Heterogeneity Identified by Stereo-Seq Analysis and Leiden Clustering Analysis

**Figure 3:**
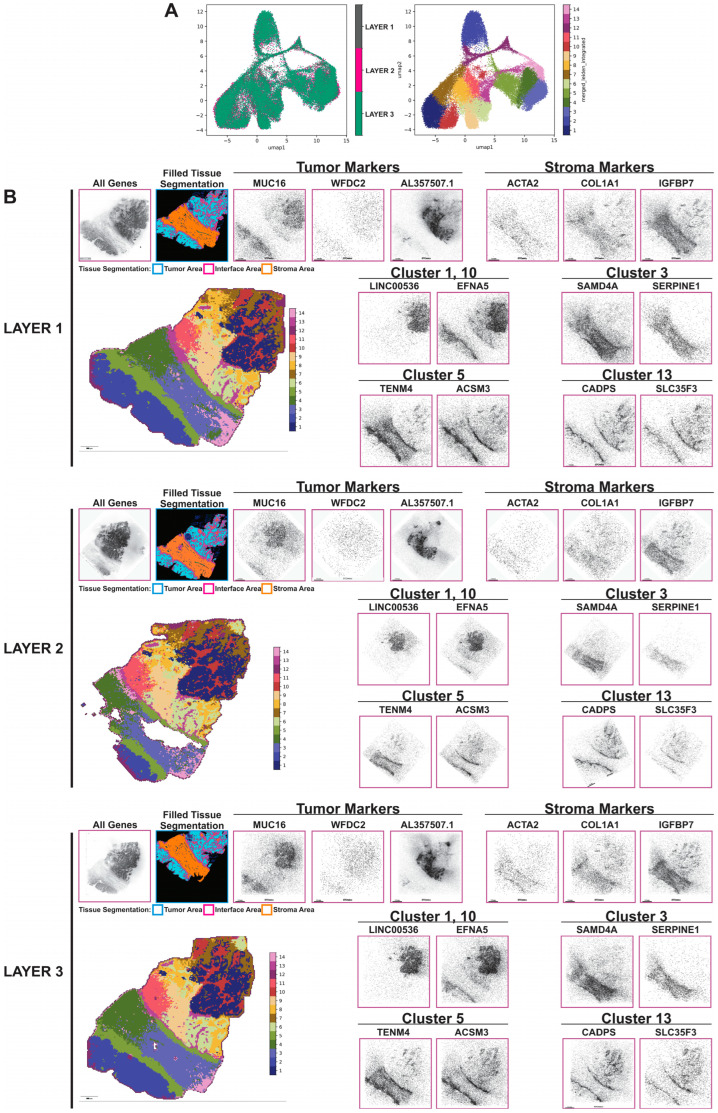
Non-targeted spatially resolved transcriptomic analysis. (**A**) UMAPs generated after Stereo-seq analysis for HGSOC sample. (**B**) Quantitative Leiden cluster analysis and qualitative images for most differentiated genes exported from STereoMap software for each layer and overlaid to seqIF images through Visiopharm software version 2023.09 x64 (tissue segmentation overlay shown in the second panel). Scale bar is 1.5 mm.

Stereo-seq identified a collection of markers in the HGSOC sample that are associated with the tumor and stromal compartments, and the tumor–stroma interface identified through tissue segmentation, and some of them are depicted in Figure 3. We utilize the uniform manifold approximation and projection (UMAP) dimensionality reduction method to provide a 2d representation of the relationships between the data in the Stereo-seq dataset. The left figure in (Figure 3A) indicates that all three sections fall within the same UMAP space, indicating the likely absence of major batch effects between transcriptomic runs between the individual tissue layers. The right panel of Figure 3A reveals that the Leiden clustered data, plotted against UMAP coordinates, shows how the Leiden clusters separate well from one another in the UMAP. MUC16 and WFDC2 are known markers for HGSOC cells and Stereo-seq data demonstrated the expression of these two genes in the tumor compartment of the HGSOC sample. AL357507.1, a long non-coding RNA (lncRNA) which has been shown to be associated with advanced stage clear cell renal cell carcinoma and osteosarcoma metastasis [34,35], was found to be highly expressed in a particular cluster of cancer cells in the HGSOC tissue. In the stromal area, high levels of ACTA2 (aSMA) and COL1A1 were detected in the stromal region as previously reported [22]. In addition, IGFBP7, a tumor associated stroma markers with growth-promoting effects in colon cancer through a paracrine tumor -stroma interaction [36], was highly expressed in the stromal compartment. Finally, CADPS, a novel neural and endocrine-specific cytosolic protein required for the Ca^2+^-regulated exocytosis of secretory vesicles [37,38], was highly expressed by HGSOC cells particularly in the tumor–stroma interface. Quantitative Leiden cluster analysis was then performed based on unsupervised clustering of the Stereo-seq transcriptomic data. High levels of heterogeneity within both the epithelial, stromal, and interface compartments with multiple subclusters were identified (Figure 3B and Supplementary Data S1). For example, LINC00536 and EFNA5 were highly expressed in clusters 1 and 10, which represented two sub-clusters of cancer cells. SADMA4 and SERPINE1 were highly expressed in cluster 3, one of the sub-clusters of the stroma compartment. TENM4 and ACSM3, as well as CADPS and SLC35F3, were highly expressed in clusters 5 and 13, respectively, which represented two different sub-clusters in the tumor–stroma interface area. While it is interesting that most of these genes have not been implicated in the development of the two diseases, their expression patterns need to be validated using multiplex seqIF and correlated with clinical outcomes before determining their roles in the pathogenesis of both diseases. 

Stereo-seq also identified a collection of markers in the AEH sample that are associated with the hyperplasia glandular epithelium and associated stroma in the endometrium and smooth muscle cells in the myometrium (Figure S2). High levels of heterogeneity were identified by quantitative Leiden clustering analysis, particularly in the endometrium. A total of 14 clusters were identified, and they were quite consistent among the 3 sections. Markers that are specific of each cluster were identified. For example, NPAS3 and NELL1 were highly expressed in cluster 3, which represented the glandular epithelial cells in the endometrium. IGF1 and ADAM12 were highly expressed in cluster 5, which represented the stroma surrounding the endometrial glands. MECOM and RHEX were highly expressed in cluster 6, which represented the luminal area within the endometrial glands. Finally, SLP1 and ERBB4 were highly expressed in cluster 12, which represented stroma adjacent to the myometrium (Figure S2 and Supplementary Data S2).

### 3.3. Mass Spectrometry Imaging Analysis

**Figure 4:**
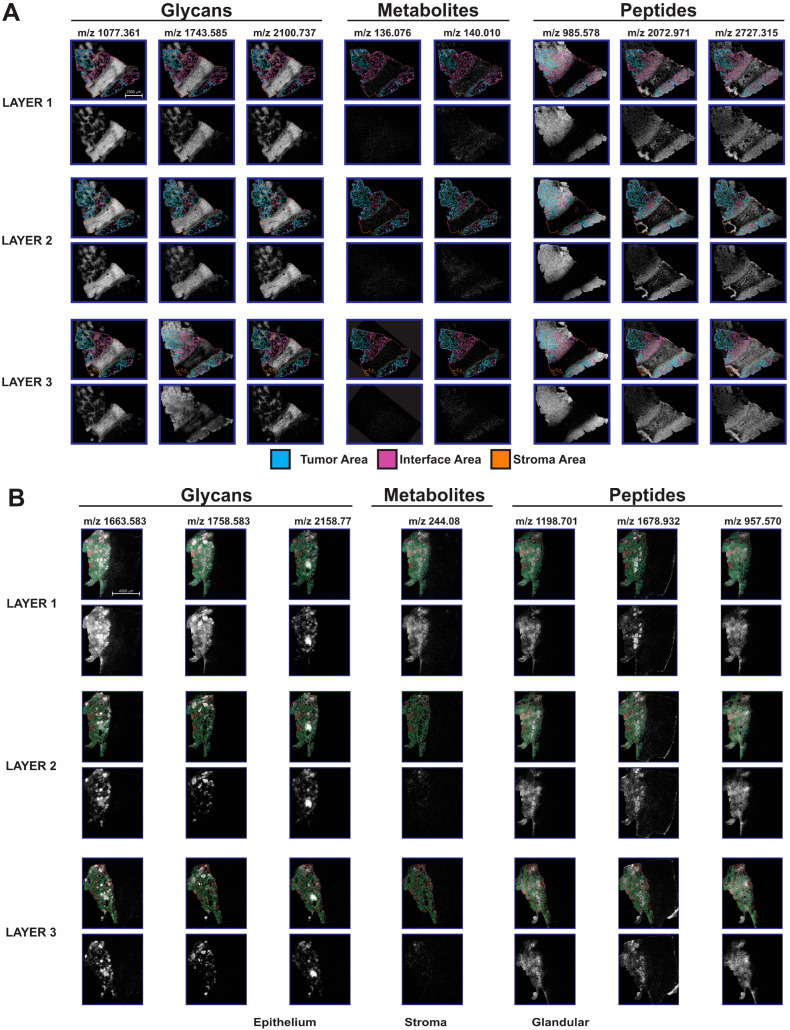
Non-targeted metabolomics, glycan, and tryptic peptide analysis by mass spectrometry imaging (MSI). (**A**,**B**) Localization of glycans, metabolites and tryptic peptides that correlates with segmented tissue areas for HGSOC (**A**) and AEH (**B**) samples. Scale bars 2500µm (**A**), 4000µm (**B**).

At each layer in the section stack, mass spectrometry imaging was preformed to map localization of metabolites, glycans, and tryptic peptides, sequentially, from the same tissue section. After loading each dataset into SCiLS Lab (Bruker Inc., Billerica, MA, USA), features were manually selected from the average spectrum resulting in 948 metabolites, 164 glycans, and 586 peptides detected. Metabolites were selected with a peak width of 15 ppm while glycans and peptides were selected with a peak width of 10 ppm. Biomolecule images from each dataset were evaluated for features that correlated with segmented regions determined by COMET analysis of serial sections. Figure 4 highlights some of these localized features. Within the metabolite data, *m*/*z* 136.076 was found to be most abundant in stroma and interface areas, while *m*/*z* 140.010 was more uniformly distributed but showed a slight decrease in stroma of HGSOC sample (Figure 4A). Three N-linked glycans at *m*/*z* 1077.361 and 2100.737 (core fucosylated and sialated) showed high abundance in the areas of stroma, and interface with lower abundance in tumors showed high abundance in areas of stroma and interface with lower abundance in tumor areas while *m*/*z* 1743.585 (high mannose) showed higher abundance in tumor and interface (Figure 4A). Finally, tryptic digestion imaging was performed on the same sections. Selected ion images show *m*/*z* 958.578 highly abundant in areas of tumor and interface but absent from stroma. A peptide at *m*/*z* 2072.971 was found to be increased in tumor just outside the main band of stroma in the sections with low abundance detected across nearly all sections. The peptide at *m*/*z* 2727.315 showed the highest levels in stroma and tumor immediately adjacent to the large stromal band but had overall more diffuse localization across the sections throughout all 3 layers.

Similarly, the AEH sample was evaluated for biomolecules that localized to the glandular portion of the sections (Figure 4B). A metabolite at *m*/*z* 244.080 was detected throughout this region with highest abundance in a subset of the glands. Three example glycan images are shown at *m*/*z* 1663.583 (complex type), 1758.583 (unknown), and 2158.770 (fucosylated), respectively, with all displaying highest abundance within the lumen of glands. However, differences are observed between the 3 glycans in which specific glands have the highest abundance of each glycan. Within the peptide data for this sample, *m*/*z* 1198.701 (actin) was found to be most abundant in epithelial areas, while *m*/*z* 1778.932 has highest expression levels within the glands, similar to patterns observed with the glycans. This peptide was also observed in the non-glandular part (right side) of the sections. The peptide at *m*/*z* 957.570 had a more diffuse distribution within the glandular region of the tissue than the other two peptides.

### 3.4. Multi-Modality Data Integration

**Figure 5:**
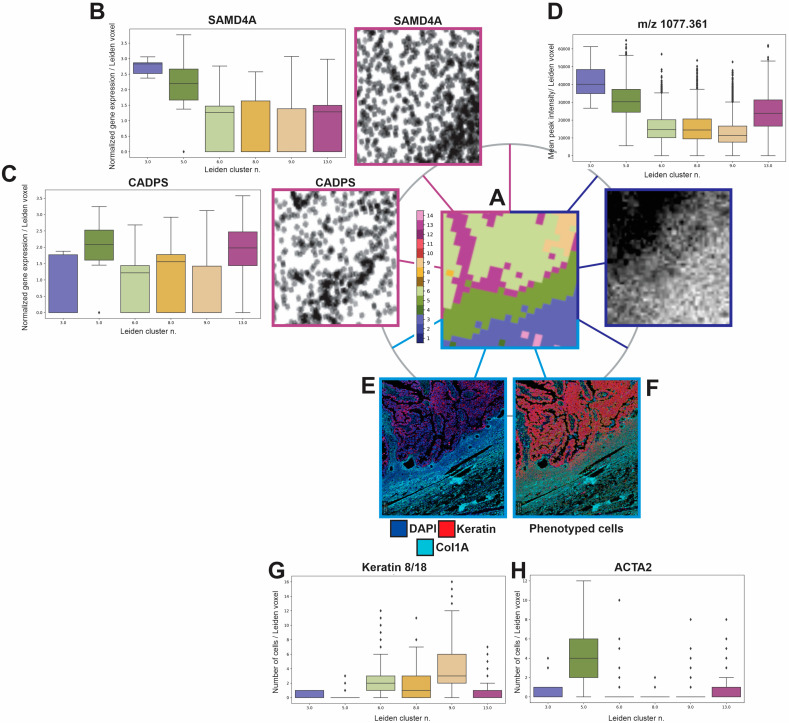
Demonstration at the region of interest level of the alignment of Leiden clusters with MSI and Comet data. (**A**) indicates the presence of Leiden clusters within one ROI, which is then indexed for alignment to the other datasets. (**B**) SAMD4A normalized gene expression (image) is measured for every bun50 ‘voxel’ within the Leiden cluster regions, showing differential expression across discrete Leiden clusters, primarily in stromal regions. (**C**) CADPS-normalized gene expression (image) shows higher prevalence in Leiden clusters 5 and 13, corresponding to the tumor:stromal interface. In (**D**), following alignment using the registered indexed cluster, a single peak from the MSI imaging dataset (*m*/*z* 1077.361), the MSI pixels corresponding to each bin50 Leiden cluster ‘voxel’ are quantitated in the box plot, showing a similar enhancement of peak intensity matching with stromal and tumor:stromal interface regions of the tissue. In (**E**), the DAPI (nuclei), Keratin (tumor epithelium) and Col1A (tumor stroma) immunostaining is shown alongside (**F**) segmented and phenotyped cells, which are then summed within each bin50 voxel to produce (**G**) box plots indicating the mean number of Keratin+ cells within each bin50 voxel, indicating higher abundance in Leiden clusters 6, 8 and 9, corresponding to tumor enriched Leiden clusters. (**H**) shows per-voxel cell numbers for ACTA2 (aSMA), with highest abundance at the tumor:stromal interface on Leiden cluster 5.

As has been mentioned previously, the generation of Leiden clusters from Stereo-seq data, plotted on an x–y coordinate framework, permits us to interrogate the spatial relationships within the dataset itself, identifying a number of unique spatial regions that seem to match well with morphological landmarks within the histology and seqIF staining regions. In order to refine the ability to do more precise alignment and retrieval of particular regions, we instituted an additional step of enumerating each bin50 region, providing an index of unique intensities across every bin50 ‘Leiden voxel’ within the tissue space (Figure 5A). Sampling the STOmics data at bin50 produced data inherently aligned with the Leiden clusters, permitting us to interrogate the normalized gene expression values on a per-Leiden cluster basis within the same ROI shown in Figure 5A. Two representative genes, SAMD4A (Figure 5B) and CADPS (Figure 5C), alongside quantitative box plots showing the mean and variance of genes within each Leiden cluster region in the ROI, are included as an example. Generating a ‘Leiden voxel indexed image’ as shown in Figure 5A, in which each bin50 region has a unique intensity value, the resulting image was then used for alignment with the individual modalities, first by matching the edges of the tissue, followed by more refined adjustments to maximize the alignment between each subregion within the tissue. This ‘common coordinate system’ permits us to pass data between other modalities that have been aligned to it. including Figure 5D a representative MSI Glycan peak (*m*/*z* 1077.361) in the same region of interest, in the same orientation as the Leiden clustered image. Quantitative box plots show the mean and orientation as the Leiden clustered image. Quantitative box plots show the mean and variance in peak intensity across the Leiden clusters ROIs. COMET data is at higher resolution than bin50 grouped data, such that several COMET pixels fit within each Leiden cluster ‘voxel’. Utilizing tissue alignment algorithms to align the COMET data with the Leiden cluster data, similarities were noted between the tissue morphology and molecular characteristics, and the Leiden cluster-informed differential genes being expressed in these regions. Figure 5E shows representative channels of COMET data, indicating epithelial and stromal regions within the tissue. Figure 5F shows phenotyped cells within this same region, which can then be summed across each Leiden cluster voxel that is present. The resulting graphs in Figure 5G show the presence of keratin, indicative of epithelial or cancer tissue, revealing which Leiden clusters are primarily epithelial in nature, and Figure 5H, the number of ACTA2 (aSMA) positive cells, which tend to be more prevalent in stromal tissue regions. This permits us to identify stromal or tumor features that might correspond with particular subsets of gene expression, such as SAMD4A and *m*/*z* 1077.361, which show higher expression in stromal-associated Leiden clusters, and CADPS, which appears at the tumor:stromal interface, corresponding to Leiden clusters 13 and 5. This cross-modality comparison permits us to either interrogate the differential gene expression on a per-cell group basis, or alternatively to re-sample the STOmics dataset at lower bin sizes, in order to identify transcripts aligned with individual cells within the dataset itself.

## 4. Discussion

In this study, we developed an analytical pipeline which integrated 3D spatially resolved data generated from non-targeted mass spectrometry imaging (glycans, metabolites, and peptides), Stereo-seq (spatial transcriptomics) and targeted seqIF (IHC proteomics) using FFPE sections prepared from a HGSOC and an AEH precancerous sample. The dataset we are describing here has many dimensions. A 3D piece of tissue was first sectioned and then split across multiple analytical modalities. Those three dimensions were then indexed on a thirty-plex high resolution CyIF protein immunostaining platform (COMET), which outputs a high resolution (250 nm) sequence of images, exported in a pyramidal ome.tiff format. Added on to those tens of protein markers, we add thousands of mass spectrometry peaks, corresponding to glycans, metabolites and peptides existing within the tissue. And then added to those thousands of spatial and molecular dimensions, we add the approximately 30,000 human genes and additionally more non-nuclear genetic reads (mitochondrial genomes, microbiome genomes). 

Integration of multiple omics platforms have been recent used to identify spatially resolved biomarkers in 2D space associated with tumor progression [25,39]. However, most of these studies utilized targeted approaches with only two platforms. In this study, we successfully used non-targeted STOmics Stereo-seq combined with seqIF to generate a 3D spatially resolved transcriptome map using FFPE sections and subsequently integrated the data with those generated from non-targeted MSI to generate a comprehensive macromolecule 3D map of the tumor tissue. This approach uses a novel version of the STOmics pipeline that permits the sampling of FFPE samples versus fresh frozen tissue. The STOmics method holds the highest resolution so far of any non-targeted assay by at least an order of magnitude, depending on how the data is binned. At the time of preparing this manuscript, 10× Visium HD (2 microns × 2 microns) has just been released and has been reported to use a bin 4 (8 microns × 8 microns). This equates to a STOmics bin 16, permitting the higher resolution assessment of genes of interest at down to bin 5 (2.5 microns × 2.5 microns) and bin 10 (5 microns × 5 microns). There is a tradeoff between bin size and collection efficiency per bin, that was recognized visually when inspecting the STOmics dataset, so for that reason we chose to set the bin size for Leiden cluster analysis on detected genes to bin50 (25 microns × 25 microns), a size that is practically quite close to the MALDI-Mass Spec image resolution (20 microns × 20 microns), permitting easy comparison of the data between those modalities; and still small enough that anywhere from one to a handful of cells might be present per bin at the protein/COMET level. 

One of the novel benefits of STOmics Stereo-seq ST technology is that, since it is untargeted, it is capable of collecting information from non-nuclear transcripts, such as mitochondrial RNA, which was detected in the assay, but not yet used for subsequent analysis. Also detected were sequences derived from the microbiome, which, while potentially quite powerful for potential future spatial analysis of the tumor microbiome, did not have sufficient read counts in the initial attempts to be able to make any conclusions regarding spatial distribution of the bacterial signatures. Given that this was one of the first applications of this technology to FFPE tissue, optimization is likely needed to develop these tools further. Also of interest is that through this analytical pipeline, the MSI data may find correlatives that could help validate the microbiota’s identification. This could be furthered by using RNAscope, an ACD application, on the Lunaphore COMET seqIF assay, a method currently in development in our laboratory. Another novel benefit in utilizing the Stereo-seq ST technology includes the ability of detecting and quantify mutated and alternative spliced transcripts in the 3D space, which have not been examined in this study.

Beside using the newly developed Stereo-seq platform, another novelty in this study is that MSI was used to generate metabolites, glycans, and tryptic peptide profiles on the same FFPE tissue section. In typical MSI experiments, only one image is collected per section of tissue, and serial sections are employed if more than one biomolecular class is to be imaged. Here, however, we have developed a method that allows us to collect 3 mass spectrometry images (metabolites, glycans, and tryptic peptides) sequentially from the same tissue section. In this way, we are able to compare different classes of molecules from the same cells as opposed to slightly different cell populations in serial sections of tissue. Additionally, since all data are collected from the same section, the separately collected datasets are inherently co-registered to each other. Using the Image Ion Mapper feature in the SCiLS Lab 2024a software (Bruker, Daltonics, Billerica, MA, USA), we can directly integrate all 3 datasets and view them simultaneously, allowing determination of the co- and differential localization of these 3 classes of biomolecules. This is of utmost importance when trying to understand cellular communication within the TIME.

Despite much recent progress, it remains challenging to integrate spatially resolved multiplexed multi-omics data, in part as success highly depends on the pathological quality of the tissue samples. Storage of tissue in FFPE blocks, permit the tissues under investigation to be cut into 3–5 mm sections, with the fixation helping to optimize tissue morphology relative to frozen sections—the fact that we could get this pipeline working forms the foundation of integrating these modalities. While they are separate tissue sections, the same cell can transverse multiple sections, as on average a cell is 20 mm in diameter, meaning one cell can be captured between 3–5 sections on average. Note that in these studies we used three groups of three sections spaced ten microns apart. This means the depth of each study plane is 9–15 microns and should contain the same cell vertically, but after an additional 10 microns transition into a new cell for the next study plane. As spatial proteomics based in IHC methods such as seqIF retain the best markers for tissue region (PanCK: Tumor, SMA: Stroma, CD31: vessel, etc.) and cell (CD4: t-cell, CD68: Macrophage, CD56: NK cell, etc.) identification. By using this technology in the central section of each study plane the analysis will use these annotations in the adjacent (above and below) sections where STOmics Stereo Seq non-targeted spatial transcriptomics and Mass Spectrometry Imaging non-targeted spatial metabolomics, glycomics, and peptidomics will collect information about these various analytes. Therefore, the key to integration is aligning the various sequential sections and passing the SeqIF annotations (tissue and cell) to non-targeted methods to allow for collection of output variables (intensity, location, morphometry, etc.). Once this is performed at the three study planes these three planes can be reassembled at both the imaging and data table level to allow for integration of the multiplex multi-omics data to create a three-dimensional cartesian coordinated map of the various samples used in this study. While this sounds straightforward the sheer number of analytes detected in this simple example study is staggering and computationally heavy. Also, by facilitating the correlation of the various analytes novel findings are possible as are novel molecular mechanisms primarily focused on cell–cell communications and interactions, specifically immune–tumor cell engagement. In this manuscript, we primarily focused on the tissue level details, specifically the tumor, interface, and stromal regions of the ovarian cancer sample building on our past research studies. However, we were able to dive into the cellular level details as indicated in the MSI and Stereo-seq data. 

It remains challenging to integrate multiple spatially resolved omics platforms with different resolution in data analysis. It is imperative when doing such an exercise to pick a coordinate system upon which to base the remaining analysis. We chose the largest dimensional dataset, the STOmics tissue array coordinates, upon the realization that the other datasets could be converted to ome.tiff formats and aligned using existing software techniques (Visiopharm commercial software version 2023.09 x64, and the combination of QuPath software version 0.5.0, Warpy software version 0.3.0 and ImageJ software version 1.54f), whereas there are not yet equivalent easy-to-use alignment methods in the scverse space (though there is a lot of innovation in this space). We feel that our efforts here represent the first demonstration of a methodology to systematically utilize dimensionality reduction and clustering in a spatially aware context, in order to resolve the necessary outputs across the tens of thousands of dimensions expressed in this dataset.

Given the choice of coordinate system with the STOmics data, the next choice was what element of the STOmics data we should use as an ‘anchor point’ from which to tie the modalities together. Standard analytical packages on high dimensional datasets like this one rely on dimensionality reduction and clustering methods, in particular Uniform Manifold and Projection (UMAP) followed by Leiden clustering, to reduce the number of dimensions taken to assess the data into a manageable form. Interestingly, even though spatial information was not incorporated in the clustering analysis, the clusters of related genes (in gene-space), when mapped to x and y coordinates (in physical space), at the bin size that the STOmics data was sampled at. This mapping revealed a striking number of morphologically interesting features as shown in Figure 3, that seemed to match with tissue phenotype features found in the COMET dataset. We decided to generate a new ‘image’, consisting of Leiden cluster ‘voxels’ at bin50 resolution (25 mm × 25 mm), which was chosen provided a compromise between low read counts in smaller bins, and losing too much spatial information at higher bins—though re-binning and re-sampling of Leiden clusters at different resolutions could certainly be tested, though perhaps not at the resolution of the whole image. 

This Leiden cluster ‘image’ was then converted into a stack of 15 image ‘channels’, in which each ‘channel’ contained the tissue ‘voxels’ for that particular channel. We assigned each individual Leiden bin a unique number (in our tissue samples there were between 15,000 and 25,000 ‘Leiden voxels’, corresponding to a 16 bit 15 channel ‘indexing tool’ that we could then align to each subsequent modality.

We used either commercial (Visiopharm) or open source (QuPath, Warpy and ImageJ) tools to align these Leiden cluster images (consisting of x,y, channel data arranged in a tiff file), with the COMET data, and with the Mass spectrometry Glycan, Metabolite, and Peptide datasets. COMET data is stored as pyramidal ome.tiff files, and the resulting aligned data, can be rendered back for a particular region of interest, a representative region illustrating this is shown in Figure 5. 

The same Leiden cluster voxel images were aligned to the Bruker MALDI mass spectrometry data, and the subsequent combined image was then queried to identify biologically relevant regions of interest. And, with the coordinate system preserved in the voxel edges of the Leiden clusters themselves, the STOmics data is accessible by querying the relevant x and y coordinates of the AnnData object storing the Leiden cluster, either at the same bin size, or by reducing bin size to address cellular or subcellular information.

Using the methodology described above, we demonstrated that we can simultaneously query various cellular neighborhoods in both tissue samples using spatially resolved imaging data in a two- and three-dimensional spaces. We also showed the collection of spatially resolved analytes in a voxel (3D pixel) across serial sections of the tissues.

There are several limitations to this study. First, only one HGSOC and one AEH samples were used in this study. More samples are needed to be examined by our 3D multi-omics profiling pipeline if we want to determine whether those markers identified by can be used in modulating the clinical management of patients with HGSOC and AEH. Second, we primarily focused on the tissue level details in this study, specifically the tumor, interface, and stromal regions of the HGSOC and AEH samples building on our past research studies. However, we can dive into the cellular and subcellular level details as indicated in the MSI and Stereo-seq data. Finally, the spatial distribution of the microbiomes, and its integration with the host transcriptomes and MSI data have been examined in this study. Further validation on the microbiome distribution needs to be performed to eliminate the impact on sequence-based microbiome analyses from tissue handling and regent contamination.

## 5. Conclusions

Mapping tumors and precancerous lesions is critical in personalizing medical treatments to the environments found in each patient and holds the best promise for the optimal outcome for that patient. This means an understanding of which cells are present, what their functional statuses are, and how they are energized is critical to understanding how a patient’s disease will progress and in identifying the aforementioned optimal treatment. In this manuscript, we have demonstrated an integrated multiplex multi-omics methodology to generate a three-dimensional tissue map of multiple tissues. Even in this limited example, several novel findings were identified, including new markers associated with stromal (SAMD4A) and tumor:stromal interface (CADPS) regions and *m*/*z* 1077.361, which show higher expression in stroma-associated areas from spatial transcriptomics and MSI data, respectively. By examining the 3D structure of the tissue and understanding how cellular neighborhoods are constructed, we have a more comprehensive understanding of the TIME. We have also mitigated the weaknesses of any single technology by leveraging the strengths of an adjacent technology to build a comprehensive map of the TIME. As we dive deeper into the data mapped to the central cartesian coordinate map we can further correlate these analytes with each other and with cellular functions such as movements and communications, as well as clinical outcomes and responses to therapies. This will function on multiple levels and allow for the development of not only better model systems to test novel therapies, but may also suggest novel therapies, which will improve the survival rates of cancer patients.

## Figures and Tables

**Table 1:** HTX M5 Sprayer Parameters.

	Metabolites	PNGaseF	Glycan Matrix	Trypsin	Peptide Matrix
Matrix/Enzyme	DAN	PNGaseF	CHCA	Trypsin	CHCA
Concentration (mg/mL)	10	0.1	10	0.1	10
Solvent	50% ACN	Water	70% ACN, 0.1% TFA, 10 mM AmPhos	9% ACN, 100 mM AmBic	70% ACN, 0.1% TFA, 10 mM AmPhos
Flow Rate (mL/min)	0.1	0.025	0.12	0.01	0.12
Number of Passes	4	15	3	12	4
Nozzle Temperature (°C)	60	45	75	30	75
Track Speed (mm/min)	1200	1200	1200	750	1200
Track Spacing (mm)	3	3	3	3	3
Track Pattern	CC	CC	HH	HH	HH
Nozzle Height (mm)	40	40	40	40	40

DAN—1,5-diaminonaphthalene, CHCA—α-cyano-4-hydroxycinnamic acid, ACN—acetonitrile, TFA—trifluoroacetic acid, AmPhos—ammonium phosphate, AmBic—ammonium bicarbonate, CC—crisscross, HH—horizontal-horizontal.

**Table 2:** Bruker timsTOF fleX Imaging Acquisition Parameters.

	Metabolites	Glycans	Peptides
Polarity	Negative	Positive	Positive
*m*/*z* range	50–600	700–3500	600–4500
Number of laser shots	200	300	300
Funnel 1 RF (Vpp)	75	450	450
Funnel 2 RF (Vpp)	100	500	500
Multipole RF (Vpp)	150	500	600
Collision Energy (eV)	10	10	10
Collision RF (Vpp)	500	2700	3400
Quadrupole Ion Energy (eV)	5	5	5
Transfer time (μs)	35	140	180
Pre Pulse Storage (μs)	2	14	18

**Table 3:** Summary of results from Stereo-seq N transcriptomics chip analysis.

Sample Type	Sample Name	MID under Tissue Area	Median Reads (per bin200)	Median MID (per bin200)	Median Gene types (per bin200)	Mitochondria Transcripts	Microbiome Transcripts
HGSOC	HGSOC_4	82.40%	349,016	8442	4102	<2%	104,870
	HGSOC_9	66.80%	489,331	6193	3122	<2%	52,250
	HGSOC_14	77.70%	504,198	9964	4396	<2%	42,360
AEH	AEH_4	59.68%	253,798	9401	4169	<2%	35,650
	AEH_9	66.70%	145,040	9055	4000	<2%	24,980
	AEH_14	74.18%	159,337	10,224	4345	<2%	40,300

HGSOC: High grade serous ovarian cancer; AEH: Atypical endometrial hyperplasia; MID: Molecular identifier.

**Table 4:** Mean gene type number per bin with different bin size.

		Mean Gene Type Number			Mean Gene Type Number		
Bin	RNA Capture Area	HGSOC_4	HGSOC_9	HGSOC_14	AEH_4	AEH_9	AEH_14
1	0.5 μm × 0.5 μm	1.2	1.16	1.25	1.2	1.21	1.21
20	10 μm × 10 μm	82.62	59.44	97.85	91.64	90.87	102
50	25 μm × 25 μm	461	333	536	505	502	561
100	50 μm × 50 μm	1501	1101	1706	1592	1586	1752
150	75 μm × 75 μm	2741	2045	3050	2816	2810	3078
200	100 μm × 100 μm	3985	3010	4362	3989	4000	4339

## Data Availability

Research data from this study will be deposited at Gene Expression Omnibus (GEO) and will be available to the public after the manuscript is accepted for publication. Resource code will be available upon request to interested researchers.

## Supplementary Materials

The following supporting information can be downloaded at: https://www.mdpi.com/article/10.3390/cancers16050846/s1, Figure S1: Multiplex sequential immunofluorescence analysis on AEH sample. Figure S2: Non-targeted spatially-resolved transcriptomics analysis. Figure S3: Stereo-seq non-host spatial transcriptomics results for HGSOC sample. Figure S4: Stereo-seq non-host spatial transcriptomics results for AEH sample. Table S1: Clinicopathological characteristics of samples used. Table S2: Primary antibodies and conditions used in the COMET analysis of high-grade serous ovarian cancer sample. Table S3: Primary antibodies and conditions used in the COMET analysis of endometrial hyperplasia sample. Table S4: Secondary antibodies and conditions used in the COMET analysis. Supplementary Data S1: Differential expressed genes for each Leiden cluster in HGSCO sample. Supplementary Data S2: Differential expressed genes for each Leiden cluster in AEH sample.

## References

[1] Abdel-Salam G.M.H., Hellmuth S., Gradhand E., Kaseberg S., Winter J., Pabst A.S., Eid M.M., Thiele H., Nürnberg P., Budde B.S. (2023). Biallelic MAD2L1BP (p31comet) mutation is associated with mosaic aneuploidy and juvenile granulosa cell tumors. JCI Insight.

[2] Yang L., Li A., Wang Y., Zhang Y. (2023). Intratumoral microbiota: Roles in cancer initiation, development and therapeutic efficacy. Signal Transduct. Target. Ther..

[3] Au Yeung C.L., Co N.N., Tsuruga T., Yeung T.L., Kwan S.Y., Laung C.S., Li Y., Lu E.S., Kwan K., Wong K.-K. (2016). Exosomal transfer of stroma-derived miR21 confers paclitaxel resistance in ovarian cancer cells through targeting APAF1. Nat. Commun..

[4] Kaymak I., Williams K.S., Cantor J.R., Jones R.G. (2021). Immunometabolic Interplay in the Tumor Microenvironment. Cancer Cell..

[5] Bhai P., Turowec J., Santos S., Kerkhof J., Pickard L., Foroutan A., Breadner D., Cecchini M., Levy M.A., Stuart A. (2023). Molecular profiling of solid tumors by next-generation sequencing: An experience from a clinical laboratory. Front. Oncol..

[6] Weinstein J.N., Collisson E.A., Mills G.B., Shaw K.R.M., Ozenberger B.A., Ellrott K., Shmulevich I., Sander C., Stuart J.M., The Cancer Genome Atlas Research Network (2013). The Cancer Genome Atlas Pan-Cancer analysis project. Nat. Genet..

[7] Schumacher K., Haensch W., Roefzaad C., Schlag P.M. (2001). Prognostic significance of activated CD8(+) T cell infiltrations within esophageal carcinomas. Cancer Res..

[8] Sato E., Olson S.H., Ahn J., Bundy B., Nishikawa H., Qian F., Jungbluth A.A., Frosina D., Gnjatic S., Ambrosone C. (2005). Intraepithelial CD8+ tumor-infiltrating lymphocytes and a high CD8+/regulatory T cell ratio are associated with favorable prognosis in ovarian cancer. Proc. Natl. Acad. Sci. USA.

[9] Kondratiev S., Sabo E., Yakirevich E., Lavie O., Resnick M.B. (2004). Intratumoral CD8+ T lymphocytes as a prognostic factor of survival in endometrial carcinoma. Clin. Cancer Res..

[10] Wu F., Fan J., He Y., Xiong A., Yu J., Li Y., Zhang Y., Zhao W., Zhou F., Li W. (2021). Single-cell profiling of tumor heterogeneity and the microenvironment in advanced non-small cell lung cancer. Nat. Commun..

[11] Gonzalez-Silva L., Quevedo L., Varela I. (2020). Tumor Functional Heterogeneity Unraveled by scRNA-seq Technologies. Trends Cancer.

[12] Zhang D., Zhang Y., Xia S., Chen L., Xu W., Huo L., Huang D., Shen P., Yang C. (2023). Single-cell RNA sequencing reveals neurovascular-osteochondral network crosstalk during temporomandibular joint osteoarthritis: Pilot study in a human condylar cartilage. Heliyon.

[13] Nofech-Mozes I., Soave D., Awadalla P., Abelson S. (2023). Pan-cancer classification of single cells in the tumour microenvironment. Nat. Commun..

[14] Giesen C., Wang H.A.O., Schapiro D., Zivanovic N., Jacobs A., Hattendorf B., Schüffler P.J., Grolimund D., Buhmann J.M., Brandt S. (2014). Highly multiplexed imaging of tumor tissues with subcellular resolution by mass cytometry. Nat. Methods.

[15] Seeley E.H., Liu Z., Yuan S., Stroope C., Cockerham E., Rashdan N.A., Delgadillo L.F., Finney A.C., Kumar D., Das S. (2023). Spatially Resolved Metabolites in Stable and Unstable Human Atherosclerotic Plaques Identified by Mass Spectrometry Imaging. Arterioscler. Thromb. Vasc. Biol..

[16] Stickels R.R., Murray E., Kumar P., Li J., Marshall J.L., Di Bella D.J., Arlotta P., Macosko E.Z., Chen F. (2021). Highly sensitive spatial transcriptomics at near-cellular resolution with Slide-seqV2. Nat. Biotechnol..

[17] Moncada R., Barkley D., Wagner F., Chiodin M., Devlin J.C., Baron M., Hajdu C.H., Simeone D.M., Yanai I. (2020). Integrating microarray-based spatial transcriptomics and single-cell RNA-seq reveals tissue architecture in pancreatic ductal adenocarcinomas. Nat. Biotechnol..

[18] Clift C.L., Drake R.R., Mehta A., Angel P.M. (2021). Multiplexed imaging mass spectrometry of the extracellular matrix using serial enzyme digests from formalin-fixed paraffin-embedded tissue sections. Anal. Bioanal. Chem..

[19] Black S., Phillips D., Hickey J.W., Kennedy-Darling J., Venkataraaman V.G., Samusik N., Goltsev Y., Schürch C.M., Nolan G.P. (2021). CODEX multiplexed tissue imaging with DNA-conjugated antibodies. Nat. Protoc..

[20] Wu Y., Cheng Y., Wang X., Fan J., Gao Q. (2022). Spatial omics: Navigating to the golden era of cancer research. Clin. Transl. Med..

[21] Zhu Y., Ferri-Borgogno S., Sheng J., Yeung T.-L., Burks J.K., Cappello P., Jazaeri A.A., Kim J.-H., Han G.H., Birrer M.J. (2021). SIO: A Spatioimageomics Pipeline to Identify Prognostic Biomarkers Associated with the Ovarian Tumor Microenvironment. Cancers.

[22] Ferri-Borgogno S., Zhu Y., Sheng J., Burks J.K., Gomez J.A., Wong K.K., Wong S.T., Mok S.C. (2023). Spatial Transcriptomics Depict Ligand-Receptor Cross-talk Heterogeneity at the Tumor-Stroma Interface in Long-Term Ovarian Cancer Survivors. Cancer Res..

[23] Heindl A., Lan C., Rodrigues D.N., Koelble K., Yuan Y. (2016). Similarity and diversity of the tumor microenvironment in multiple metastases: Critical implications for overall and progression-free survival of high-grade serous ovarian cancer. Oncotarget.

[24] Arora R., Cao C., Kumar M., Sinha S., Chanda A., McNeil R., Samuel D., Arora R.K., Matthews T.W., Chandarana S. (2023). Spatial transcriptomics reveals distinct and conserved tumor core and edge architectures that predict survival and targeted therapy response. Nat. Commun..

[25] Hu T., Allam M., Cai S., Henderson W., Yueh B., Garipcan A., Ievlev A.V., Afkarian M., Beyaz S., Coskun A.F. (2023). Single-cell spatial metabolomics with cell-type specific protein profiling for tissue systems biology. Nat. Commun..

[26] Kuett L., Catena R., Özcan A., Plüss A., Ali H.R., Al Sa’d M., Alon S., Aparicio S., Battistoni G., Balasubramanian S. (2022). Three-dimensional imaging mass cytometry for highly multiplexed molecular and cellular mapping of tissues and the tumor microenvironment. Nat. Cancer.

[27] Rivest F., Eroglu D., Pelz B., Kowal J., Kehren A., Navikas V., Procopio M.G., Bordignon P., Pérès E., Ammann M. (2023). Fully automated sequential immunofluorescence (seqIF) for hyperplex spatial proteomics. Sci. Rep..

[28] Bankhead P., Loughrey M.B., Fernández J.A., Dombrowski Y., McArt D.G., Dunne P.D., McQuaid S., Gray R.T., Murray L.J., Coleman H.G. (2017). QuPath: Open source software for digital pathology image analysis. Sci. Rep..

[29] Chiaruttini N., Burri O., Haub P., Guiet R., Seitz A. (2022). An open-source whole slide image registration workflow at cellular precision using Fiji, QuPath and Elastix. Prontiers Comp. Sci..

[30] Chen A., Liao S., Cheng M., Ma K., Wu L., Lai Y., Qiu X., Yang J., Xu J., Hao S. (2022). Spatiotemporal transcriptomic atlas of mouse organogenesis using DNA nanoball-patterned arrays. Cell.

[31] Chen A., Sun Y., Lei Y., Li C., Liao S., Meng J., Bai Y., Liu Z., Liang Z., Zhu Z. (2023). Single-cell spatial transcriptome reveals cell-type organization in the macaque cortex. Cell.

[32] Dobin A., Davis C.A., Schlesinger F., Drenkow J., Zaleski C., Jha S., Batut P., Chaisson M., Gingeras T.R. (2013). STAR: Ultrafast universal RNA-seq aligner. Bioinformatics.

[33] Powers T.W., Neely B.A., Shao Y., Tang H., Troyer D.A., Mehta A.S., Haab B.B., Drake R.R. (2014). MALDI imaging mass spectrometry profiling of N-glycans in formalin-fixed paraffin embedded clinical tissue blocks and tissue microarrays. PLoS ONE.

[34] Yang F., Liu C., Zhao G., Ge L., Song Y., Chen Z., Liu Z., Hong K., Ma L. (2020). Long non-coding RNA LINC01234 regulates proliferation, migration and invasion via HIF-2alpha pathways in clear cell renal cell carcinoma cells. PeerJ.

[35] Zhang H., Chen G., Lyu X., Rong C., Wang Y., Xu Y., Lyu C. (2021). A Novel Predictive Model Associated with Osteosarcoma Metastasis. Cancer Manag. Res..

[36] Rupp C., Scherzer M., Rudisch A., Unger C., Haslinger C., Schweifer N., Artaker M., Nivarthi H., Moriggl R., Hengstschläger M. (2015). IGFBP7, a novel tumor stroma marker, with growth-promoting effects in colon cancer through a paracrine tumor-stroma interaction. Oncogene.

[37] Walent J.H., Porter B.W., Martin T.F. (1992). A novel 145 kd brain cytosolic protein reconstitutes Ca(2+)-regulated secretion in permeable neuroendocrine cells. Cell.

[38] Nestvogel D.B., Merino R.M., Leon-Pinzon C., Schottdorf M., Lee C., Imig C., Brose N., Rhee J.-S. (2020). The Synaptic Vesicle Priming Protein CAPS-1 Shapes the Adaptation of Sensory Evoked Responses in Mouse Visual Cortex. Cell Rep..

[39] Artyomov M.N., Van den Bossche J. (2020). Immunometabolism in the Single-Cell Era. Cell Metab..

