# Supplementary material for: Molecular, Metabolic, and Subcellular Mapping of the Tumor Immune Microenvironment via 3D Targeted and Non-Targeted Multiplex Multi-Omics Analyses
Source: Cancers (Basel). 2024 Feb 20;16(5):846. doi: 10.3390/cancers16050846 (PMC10930466; doi:10.3390/cancers16050846)
Supplement: Supplementary file 1 [file cancers-16-00846-s001.zip › Supplementary figures.pdf]

Supplementary figures

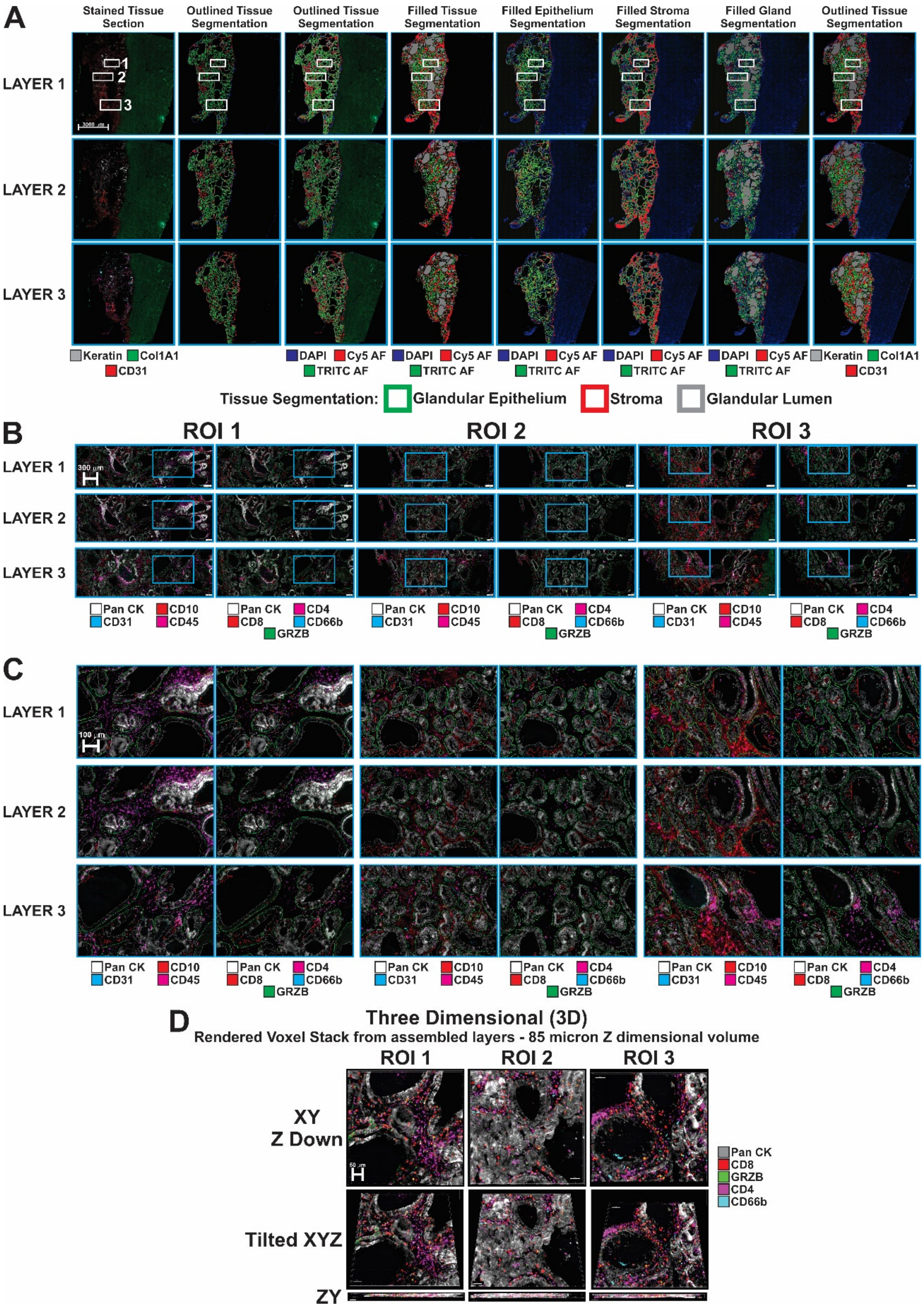

Supplementary Figure S1. Multiplex sequential immunofluorescence analysis on AEH sample. **A**. Whole tissue images overlaid with tissue segmentation mask were created based on Pan-Keratin, CD10 and αSMA markers in Visiopharm software for each layer. White boxes represent chosen ROIs shown in panel B. Scale bar is 3500 µm. **B**. Chosen ROIs from panel A, highlighting corresponding marker expression in different tissue areas. Blue square represent chosen ROIs shown in panel C. Scale bar is 300µm. **C**. Chosen ROIs from panel B, showing differential expression of main immune markers. Scale bar is 100µm. **D**. 3D reconstruction through rendered voxel stack from assembled layers 1-3. Scale bar is 50µm.

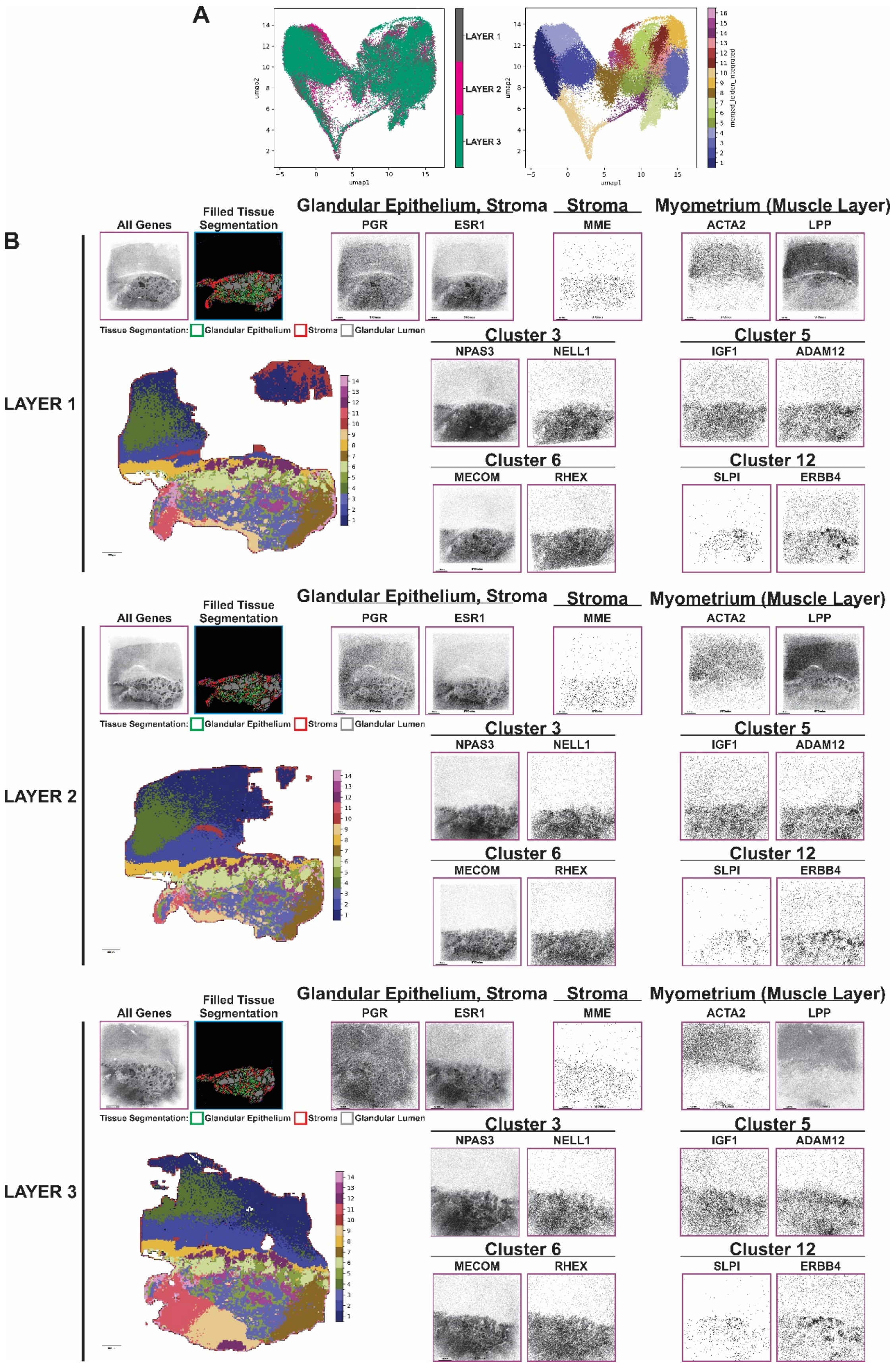

Supplementary Figure S2. Non-targeted spatially-resolved transcriptomics analysis. **A**. UMAPs generated after stereo-seq analysis for AEH sample. **B**. Quantitative Leiden cluster analysis and qualitative images for most differentiated genes exported from STereoMap software for each layer and overlaid to seqIF images through Visiopharm software (tissue segmentation overlay shown in the second panel). Scale bar is 1.5 mm.

A

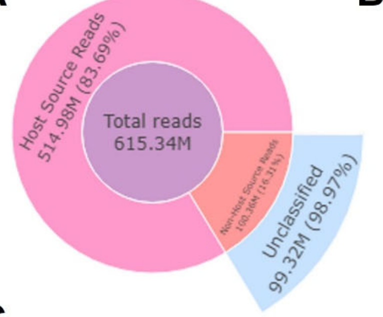

B

Taxonomic Classification of non-host reads

Non-host source reads	100.3M
Bacteria, Fungi and Viruses MIDs	52,251
Bacteria, Fungi and Viruses Duplication	596,159
Other Microbes or Host-Suspicious	389,445
Unclassified Reads	99,320,624

C

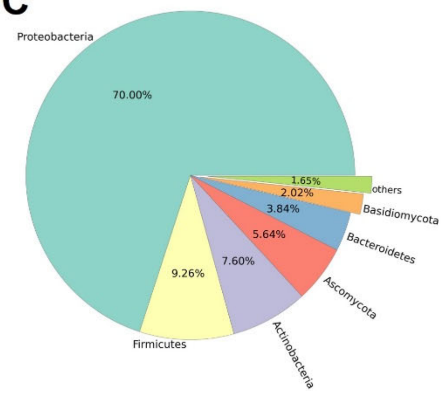

Microbes proportion (Phylum)

Phylum	MID Count	Percentage
p__Proteobacteria	35,528	70.0%
p__Firmicutes	4,700	9.26%
p__Actinobacteria	3,857	7.6%
p__Ascomycota	2,863	5.64%
p__Bacteroidetes	1,949	3.84%
p__Basidiomycota	1,023	2.02%
p__Cyanobacteria	245	0.48%
p__Artverviricota	124	0.24%
p__Deinococcus-Thermus	102	0.2%
p__Planctomycetes	81	0.16%

D

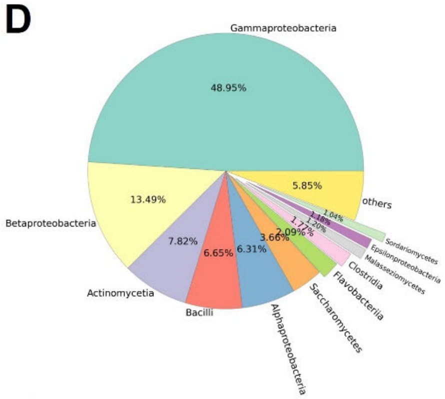

Microbes proportion (Class)

Class	MID Count	Percentage
c__Gammaproteobacteria	23,816	48.95%
c__Betaproteobacteria	6,563	13.49%
c__Actinomycetia	3,803	7.82%
c__Bacilli	3,237	6.65%
c__Alphaproteobacteria	3,069	6.31%
c__Saccharomycetes	1,782	3.66%
c__Flavobacteriia	1,019	2.09%
c__Clostridia	861	1.77%
c__Malasseziomycetes	583	1.2%
c__Epsilonproteobacteria	575	1.18%

E

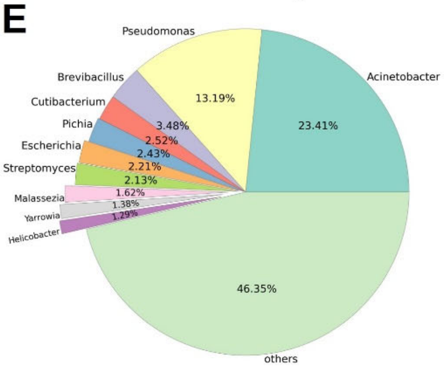

Microbes proportion (Genus)

Genus	MID Count	Percentage
g__Acinetobacter	8,409	23.41%
g__Pseudomonas	4,738	13.19%
g__Brevibacillus	1,252	3.48%
g__Cutibacterium	904	2.52%
g__Pichia	872	2.43%
g__Escherichia	794	2.21%
g__Streptomyces	764	2.13%
g__Malassezia	583	1.62%
g__Yarrowia	497	1.38%
g__Helicobacter	463	1.29%

Supplementary Figure S3. Stereo-seq non-host spatial transcriptomics results for HGSOC sample, section 9 (layer 2). **A.** Sunburst Chart of Mapping showing number of host (Number of reads that can be aligned to the host genome during denoising) and non-host (Number of reads that cannot be aligned to the host genome) reads. **B.** Taxonomic Classification of non-host reads: Bacteria, Fungi and Viruses MIDs (number of unique mRNA molecular assigned to bacteria, fungi or viruses); Bacteria, Fungi and Viruses Duplication (number of assigned reads that have been corrected due to duplicated MID); Other Microbes or Host-Suspicious (number of reads assigned to other microbes, exclude bacteria, fungi and viruses) or host; unclassified reads. **C.** The main proportion of microbes at the phylum level. **D.** The main proportion of microbes at the class level. **E.** The main proportion of microbes at the genus level.

.

A

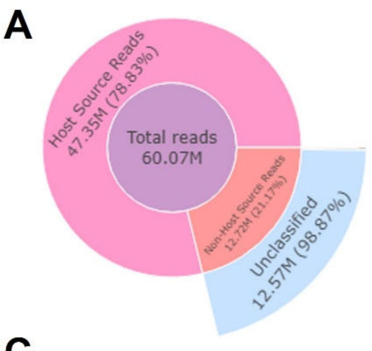

B

Taxonomic Classification of non-host reads

Non-host source reads	12.72M
Bacteria, Fungi and Viruses MIDs	24,984
Bacteria, Fungi and Viruses Duplication	46,731
Other Microbes or Host-Suspicious	71,743
Unclassified Reads	12,575,144

C

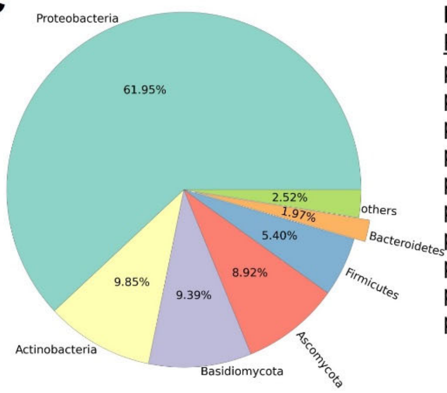

Microbes proportion (Phylum)

Phylum	MID Count	Percentage
p__Proteobacteria	15,213	61.95%
p__Actinobacteria	2,418	9.85%
p__Basidiomycota	2,306	9.39%
p__Ascomycota	2,191	8.92%
p__Firmicutes	1,326	5.4%
p__Bacteroidetes	484	1.97%
p__Kitrinoviricota	215	0.88%
p__Cyanobacteria	129	0.53%
p__Artverviricota	96	0.39%
p__Deinococcus-Thermus	33	0.13%

D

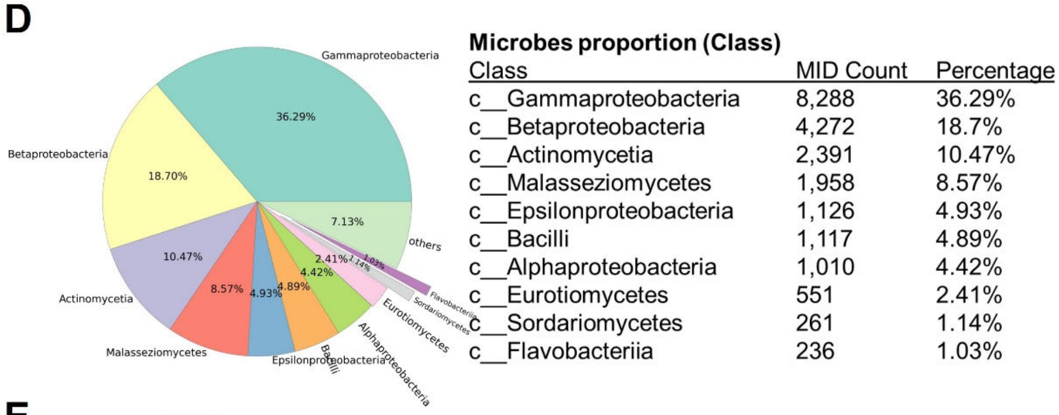

Microbes proportion (Class)

Class	MID Count	Percentage
c__Gammaproteobacteria	8,288	36.29%
c__Betaproteobacteria	4,272	18.7%
c__Actinomycetia	2,391	10.47%
c__Malasseziomycetes	1,958	8.57%
c__Epsilonproteobacteria	1,126	4.93%
c__Bacilli	1,117	4.89%
c__Alphaproteobacteria	1,010	4.42%
c__Eurotiomycetes	551	2.41%
c__Sordariomycetes	261	1.14%
c__Flavobacteriia	236	1.03%

E

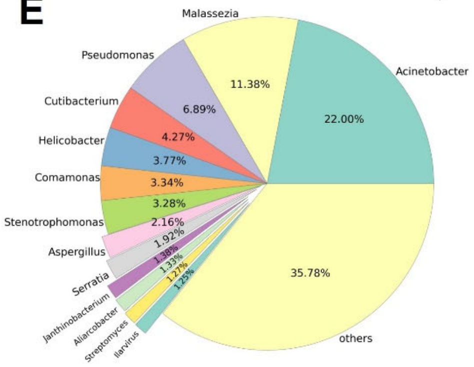

Microbes proportion (Genus)

Genus	MID Count	Percentage
g__Acinetobacter	3,785	22.0%
g__Malassezia	1,958	11.38%
g__Pseudomonas	1,185	6.89%
g__Cutibacterium	734	4.27%
g__Helicobacter	648	3.77%
g__Comamonas	574	3.34%
g__Stenotrophomonas	564	3.28%
g__Aspergillus	371	2.16%
g__Serratia	331	1.92%
g__Janthinobacterium	237	1.38%

Supplementary Figure S4. Stereo-seq non-host spatial transcriptomics results for AEH sample, section 9 (layer 2). **A.** Sunburst Chart of Mapping showing number of host (Number of reads that can be aligned to the host genome during denoising) and non-host (Number of reads that cannot be aligned to the host genome) reads. **B.** Taxonomic Classification of non-host reads: Bacteria, Fungi and Viruses MIDs (number of unique mRNA molecular assigned to bacteria, fungi or viruses); Bacteria, Fungi and Viruses Duplication (number of assigned reads that have been corrected due to duplicated MID); Other Microbes or Host-Suspicious (number of reads assigned to other microbes, exclude bacteria, fungi and viruses) or host; unclassified reads. **C.** The main proportion of microbes at the phylum level. **D.** The main proportion of microbes at the class level. **E.** The main proportion of microbes at the genus level
